# Inferior vena cava anomalies and variations: imaging and rare clinical findings

**DOI:** 10.1007/s13244-015-0431-z

**Published:** 2015-09-15

**Authors:** Bulent Petik

**Affiliations:** Department of Radiology, Adiyaman University Medical Faculty, Altinsehir Mh., 3005 Sokak No:13, 02040 Adiyaman, Turkey

**Keywords:** Inferior vena cava anomalies, Multidetector row computed tomography, Magnetic resonance imaging, IVC, Inferior vena cava variations

## Abstract

**Purpose:**

The aim of this paper is to summarize imaging findings of some frequent and infrequent inferior vena cava (IVC) anomalies and variations.

**Conclusions:**

IVC anomalies should be suspected in patients presenting with pulmonary emboli, chronic pain, and deep vein thrombosis. To correctly characterize and classify IVC anomalies and variations is of crucial importance for proper planning of surgical interventions and thus for avoiding serious complications.

***Key Points*:**

• *IVC anomalies should be suspected in patients with pulmonary emboli, pain, and venous thrombosis*.

• *Awareness of IVC anomalies and variations is crucial for clinical and surgical procedures*.

• *Unawareness of these anomalies may lead to severe and deadly complications*.

## Introduction

Anomalies of the inferior vena cava (IVC) and its variations were first described by Abernethy in 1793 in a 10-month-old child with polysplenia and dextrocardia who presented with a congenital mesocaval shunt and continuation of IVC with the azygos vein (CCA) [1–3]. The IVC develops sequentially, primarily between the sixth and eighth gestational weeks, with the formation of anastomoses and posterior regression of three paired veins: subcardinal, supracardinal, and postcardinal [2–6]. Because of this complexity in its embryogenesis, there are numerous anatomical forms and variations of IVC [2, 3, 5–8].

Anomalies of IVC are usually visualized by noninvasive imaging techniques including multidetector row computed tomography (MDCT) and magnetic resonance imaging (MRI) [2, 9]. In fact, these anomalies can be misdiagnosed as a mass lesion if variations and anomalies such as the double IVC, agenesis of infrarenal IVC, left IVC, and enlarged azygos vein are overlooked. Moreover, awareness of these variations is crucially important for cardiopulmonary surgeries. For instance, in cases with left IVC, the infrarenal placement of an IVC filter may be difficult to perform through the atransjugular approach [2–4, 10].

In this article, we present the imaging findings and rare clinical variations of IVC anomalies with associated variations and diseases.

## Imaging considerations

All CT studies were performed on a 64-channel MDCT scanner (Aquilion; Toshiba, Japan). Routine abdominal tomography scans and the portal phase images used to interpret the venous structures in the abdomen were obtained 60–70 s after the administration of 70–80 ml non-ionic iodinated contrast material and 40 ml saline at injection rates of 2.5-3 ml/s. The CT scans were evaluated on a workstation (Vitrea; Toshiba, Japan) using postprocessing techniques including maximum intensity projection, multiplanar reconstruction, and volume rendering. Routine MRI scans were obtained on a 1.5 T MR system (HDxT; General Electric, USA) following the injection of 15 ml gadolinium-based contrast material followed by 25–30 ml saline solution at a speed of 1.8 ml/s. The MR images were evaluated on a dedicated workstation (AW volume share 4; General Electric, USA) using the abovementioned postprocessing techniques.

Specific IVC variations and anomalies including associated venous variations such as continuous azygos (CA) and hemiazygos CHA) veins and other collateral pathways (deep, portal, median, and superficial) were recorded using a standardized form. 

In the current literature, there is no consensus about the classification of IVC anomalies and variations. In a study about the development of the IVC in the domestic cat performed in 1920, Huntington and McLure proposed a theoretical classification system for IVC anomalies and suggested that there could be 14 theoretical variations. In this paper, the authors stated that 11 of these 14 variants had been observed either in cat or in humans. Nevertheless, they observed other additional anomalies in humans such as abnormal development of the prerenal division of the IVC and persistence of the renal collar [11]. Today, the most frequently encountered and published anomalies include retroaortic left renal vein, left IVC, double IVC, circumaortic left renal vein, interruption of IVC with azygos and hemiazygos continuation, absence of the infrarenal IVC, and circumcaval ureter [2, 8, 12]. In general, the prevalence of IVC anomalies is reported as 0.5 % in the world [13]. In the present pictorial essay we summarize imaging findings of ten patients with frequent and infrequent IVC anomalies including absence of infrarenal IVC, left IVC, double IVC, interruption of IVC with CA, left retroaortic vein, and accessory continuous hemiazygos vein. Of these ten patients, five were men and five women with a mean age of 45 (range: 11–65 years).

### Absent infrarenal IVC

Absent infrarenal IVC is the rarest and the most striking congenital anomaly of the IVC , and can be either complete or incomplete absence with preservation of the suprarenal segment [2–4, 14, 15]. Complete absence of IVC occurs when the three paired venous systems (subcardinal, supracardinal and postcardinal systems) fail to develop properly. Complete absence of the infrarenal IVC with preservation of the suprarenal segment is highly infrequent. Absent infrarenal IVC results in the failure of development of the posterior cardinal and supracardinal veins [2]. D’Archambeau and Milner proposed that the absent infrarenal IVC is caused by the intrauterine or perinatal thrombosis of the IVC, based on the theory that a single embryological failure cannot explain the absence of infrarenal IVC. This theory is supported by numerous researchers [2, 15, 16].

Absence of IVC may be accompanied by symptoms of venous insufficiency in the lower limbs or idiopathic deep vein thrombosis (DVT), particularly in early adulthood [4, 16, 17]. Moreover, absence of IVC may also lead to insufficient blood flow, causing blood stasis in lower limbs and formation of varices, which is generally bilateral in more than half of the patients. However, this bilateral tendency contradicts the reported incidence of less than 10 % in the DVT patients presenting with a normal IVC [2, 3, 8, 9, 18, 19]. These conditions lead to the formation of collateral pathways. Eyraud divides the collateral pathways into four systems: deep, portal, median, and superficial [18].

**Fig. 1:**
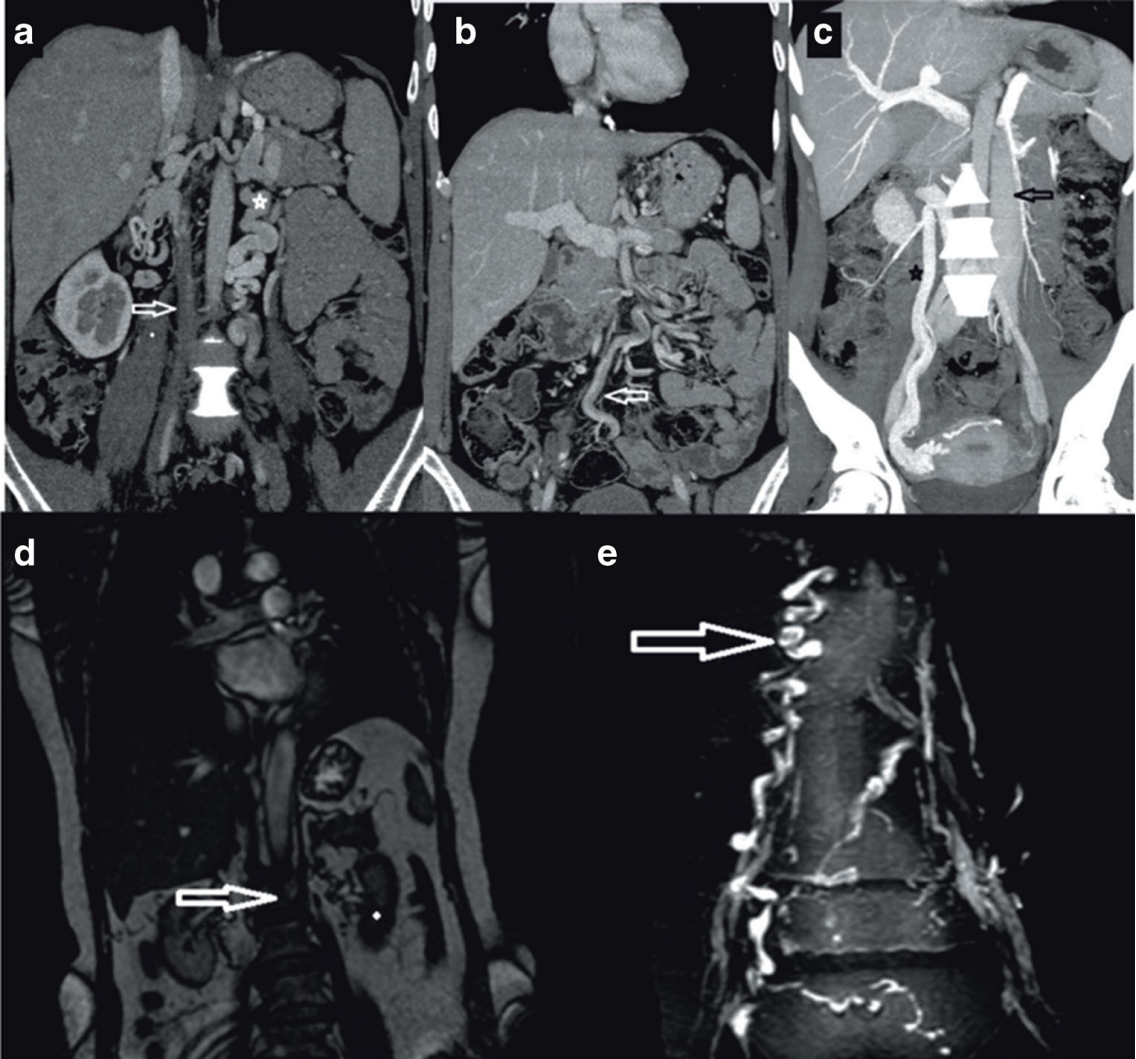
**a** Coronal maximum-intensity-projection (MIP) MDCT image showing elongated and tortuous suprarenal IVC segment (*open arrow*) progressing with intrahepatic IVC segment **b** Axial MDCT image showing concurrent CA (*asterisk*)/CHA (*arrow*) at the base of the heart **c** Coronal MDCT image showing CHA draining into CA approximately at the level of thoracal 6 (T6) (*arrow*) **d** Coronal MDCT image showing retrocrural shunts (*arrows*) secondary to IVC agenesis

In this paper we present two patients with chronic DVT and diffuse superficial varicose veins originating from the thoraco-abdominal area and extending to the diaphragmatic crura. In the first case, we encountered infrarenal agenesis and azygos continuation of IVC with active superficial, deep, and portal collateral pathways (Fig. 1). Regarding the superficial pathway, the varicose dilatations originating from the left vulvar area were draining into five veins including the right femoral vein, the epigastric vein, the internal mammary vein, the subclavian vein, and the SVC. The thoracoabdominal veins were open and draining into the lateral thoracic veins, the axillary vein, the left brachiocephalic trunk, and the SVC. The portal pathway was also being used and the paraumbilical veins were open and draining into the portal system. The ascending lumbar vein and intravertebral veins were drained by azygos and hemiazygos veins through the deep collateral pathway. Azygos and hemizygos veins combine to form the azygos continuation of the inferior vena cava (Fig. 1).

**Fig. 2:**
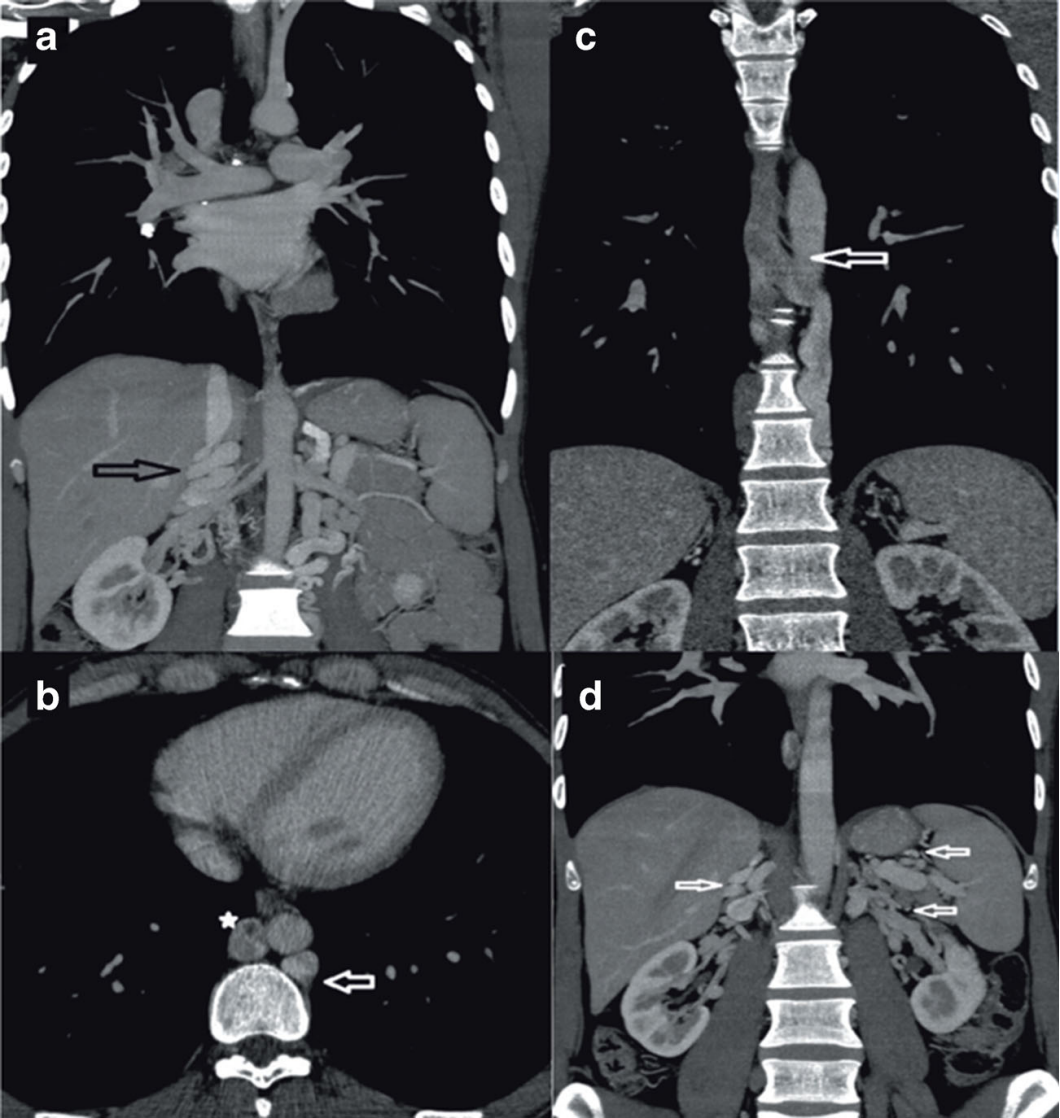
**a** Coronal MIP MDCT image showing an appearance of complete thrombosis of the infrarenal portion of IVC (on the right (*arrow*) and tortuous appearance of gonadal vein (*median pathway*) (on the *left*) (*asterisk*) **b** Coronal MIP MDCT image showing enlarged inferior hemorrhoidal vein (*portal pathway*) (*arrow*) **c** Sagittal MIP MDCT image showing ascending lumbar veins with an appearance of pseudomasses at the level of lumbar vertebra (*deep pathway*) (*arrow*) **d** Coronal MPR MRI image showing infrarenal IVC agenesis (arrow) (**e**) Coronal MPR MRI image showing enlarged epigastric vein (*superficial pathway*) (*arrow*)

In our second case, we encountered infrarenal agenesis of IVC with azygos and hemiazygos continuation. A colour Doppler US examination revealed chronic thrombosis causing near-total venous occlusion in the right main femoral and superficial femoral veins. In this patient, in addition to the superficial, portal, and deep collateral pathways, the median pathways were also active and the gonadal and pelvic veins were enlarged. The suprarenal IVC segment of this patient was tortuous and elongated but remained intact (Fig. 2).

**Fig. 3:**
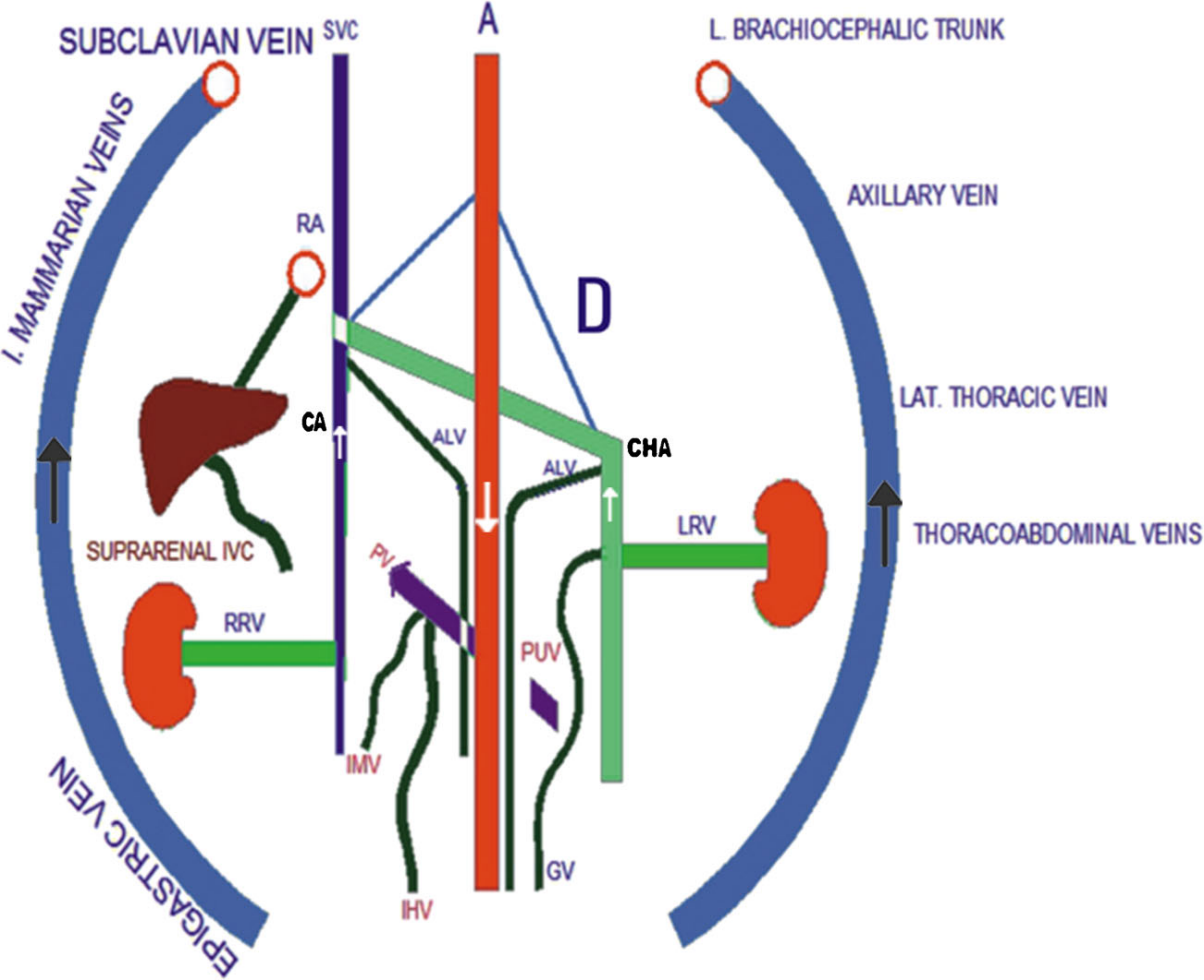
Illustration of findings of infrarenal IVC agenesis with collateral pathways including superficial (epigastric vein, internal mammarian veins, thoracoabdominal veins, lateral thoracic vein and axillary vein), portal (inferior haemorrhoidal vein (IHV), paraumbilical veins (PUV), inferior mesenteric vein (IMV)), deep (ascending lumbar vein, continuous azygos vein (CA), continuous hemiazygos vein (CHA)), and median collateral pathways (gonadal vein). (RA: right atrium; A: aorta; SVC: superior vena cava; RRV and LRV: right and left renal veins, respectively; D: diaphragm)

Figure 3 illustrates findings that may be associated with the infrarenal absence of IVC with azygos-hemiazygos continuation.

### Left IVC

**Fig. 4:**
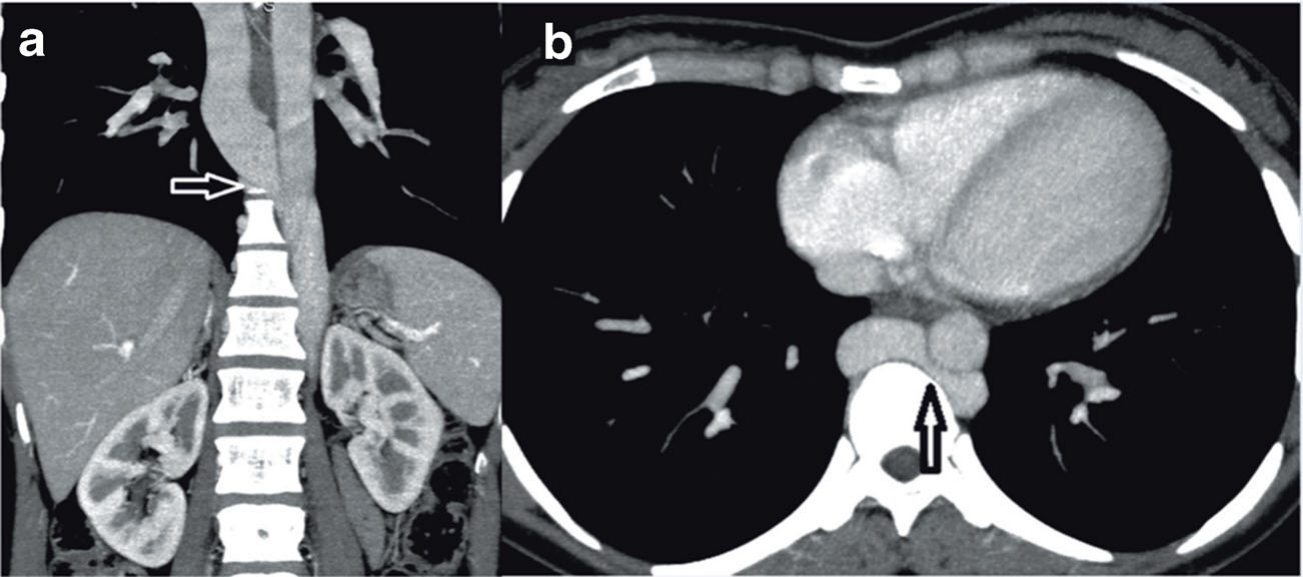
**a** Coronal MIP MDCT image showing CHA draining into CA at the level of T8-9 (*arrow*) **b** Axial MIP MDCT image showing CHA draining into CA at the level of T8-9(*arrow*)

A persistent left IVC is caused by the regression of a right supracardinal vein and the persistence of a left supracardinal vein. In this paper, we present three patients with left IVC and some unique imaging features. Typically, left IVC meets LRV, which passes anterior to the aorta in the “N fashion”, thereby joining the RV to create a normal right-sided prerenal IVC [2, 3, 7, 20]; in the literature the left IVC has a prevalence of 0.2 %-0.5 %, [3, 20]. In the cases, we present, a left IVC was draining into the CHA that was eventually joining the CA, supradiaphragmatically; combined CA-CHA was draining into SVC (Fig. 4). This pattern is not consistent with the common left IVC variations reported in the literature. Haswell et al. reported a left IVC with CHA and accessory CHA and eventually going into the SVC through the brachiocephalic route [7]. Koç et al. reported a similar variation but the drainage was promptly returning to the CAA and progressing thereafter [9]. In the literature, we were not able to find data regarding the prevalence of left CHA/CAA variations [3, 20]. We believe that left IVC with CHA and CAA is a very rare variation of IVC.

**Fig. 5:**
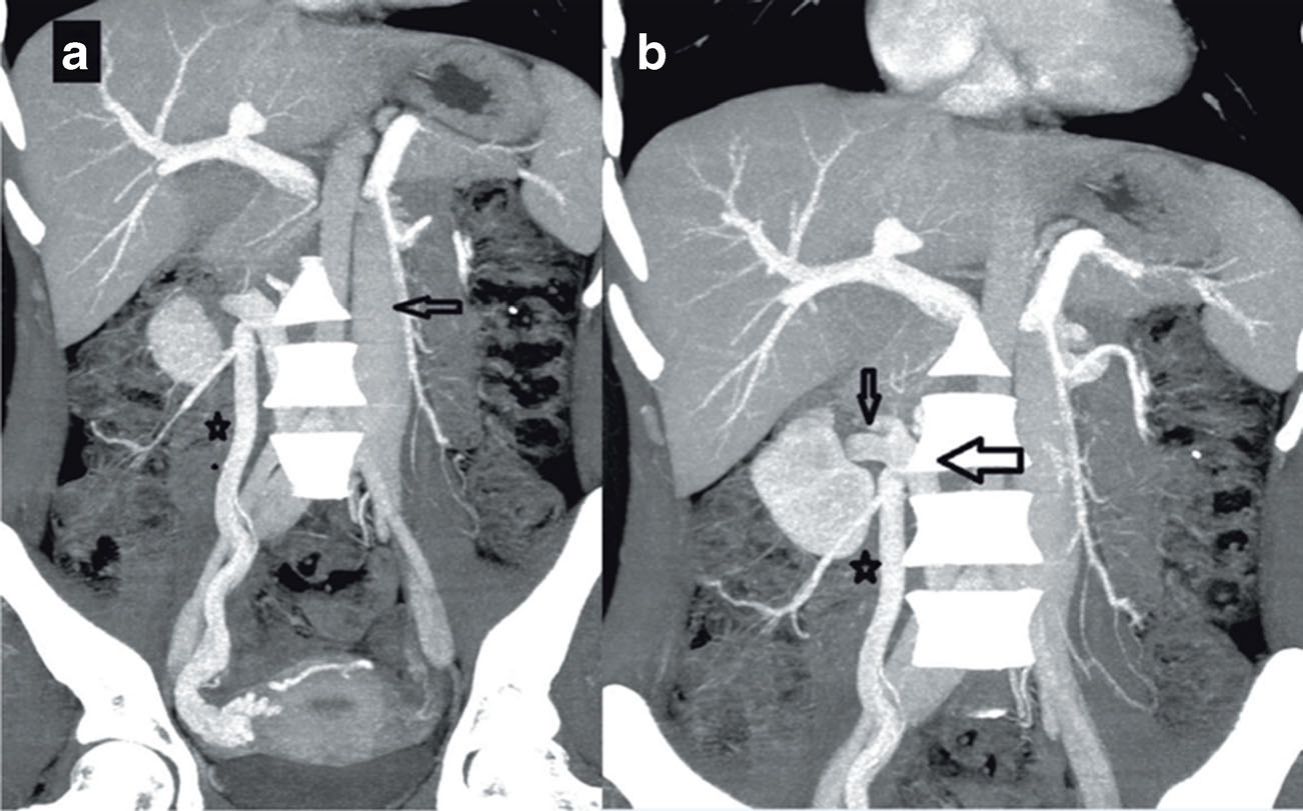
**a** Coronal MIP MDCT image showing left IVC (*arrow*) associated with dilated right gonadal vein (on the *right*) (*asterisk*) **b** Coronal MIP MDCT image showing RRV (*small arrow*) draining into the retroaortic left IVC (large arrow) in the paravertebral area, the dilated right gonadal vein is marked with an asterix

In the first of our three patients with a left IVC, the right gonadal vein was draining into the right renal vein (RRV); the right renal vein was retroaortic and draining into the left IVC. The left renal vein (LRV), was draining directly into the left IVC, (Fig. 5). A Color Doppler Ultrasound (CDUS) detected venous insufficiency in the common femoral vein (CFV), the saphena magma (SM), and the saphenofemoral junction. Diffuse superficial varicose veins passing posterior to the diaphragmatic crura were detected.

**Fig. 6:**
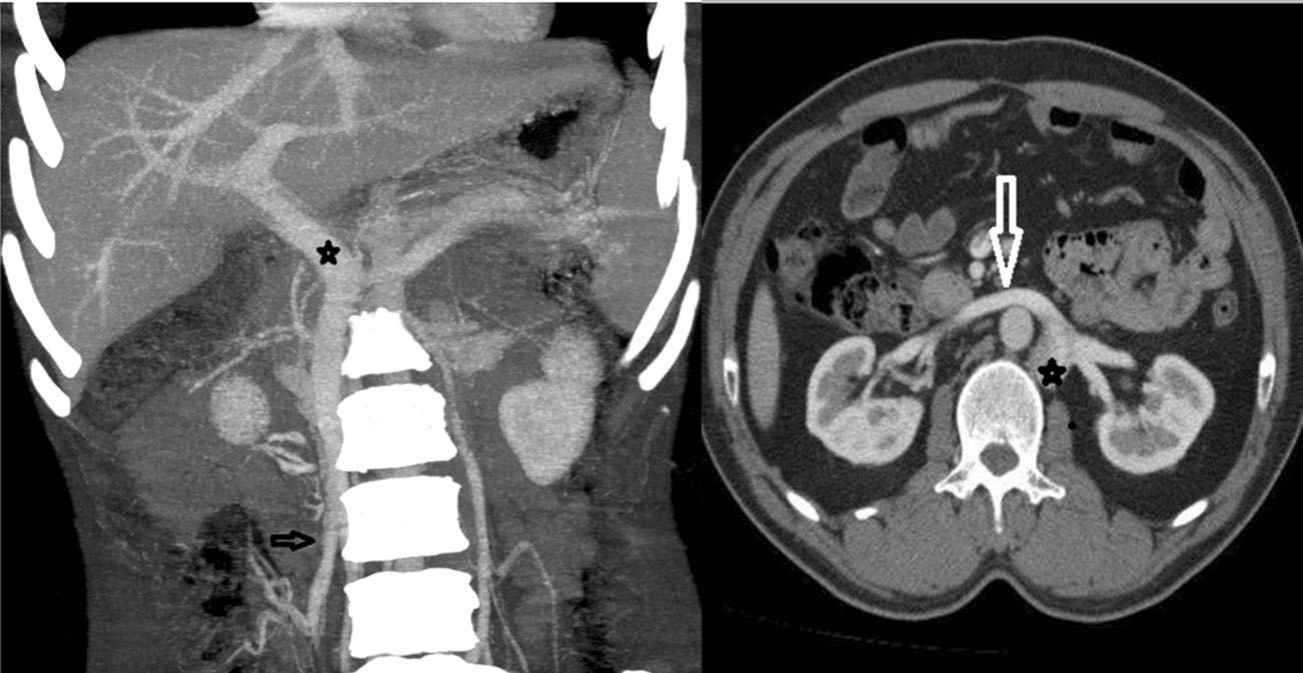
**a** Coronal MPR MDCT image showing enlarged inferior mesenteric vein (*arrow*) draining into the portal vein (*asterisk*) **b** Axial MDCT image showing RRV (*arrow*) draining into the left IVC (*asterix*) anterior to the aorta

**Fig. 7:**
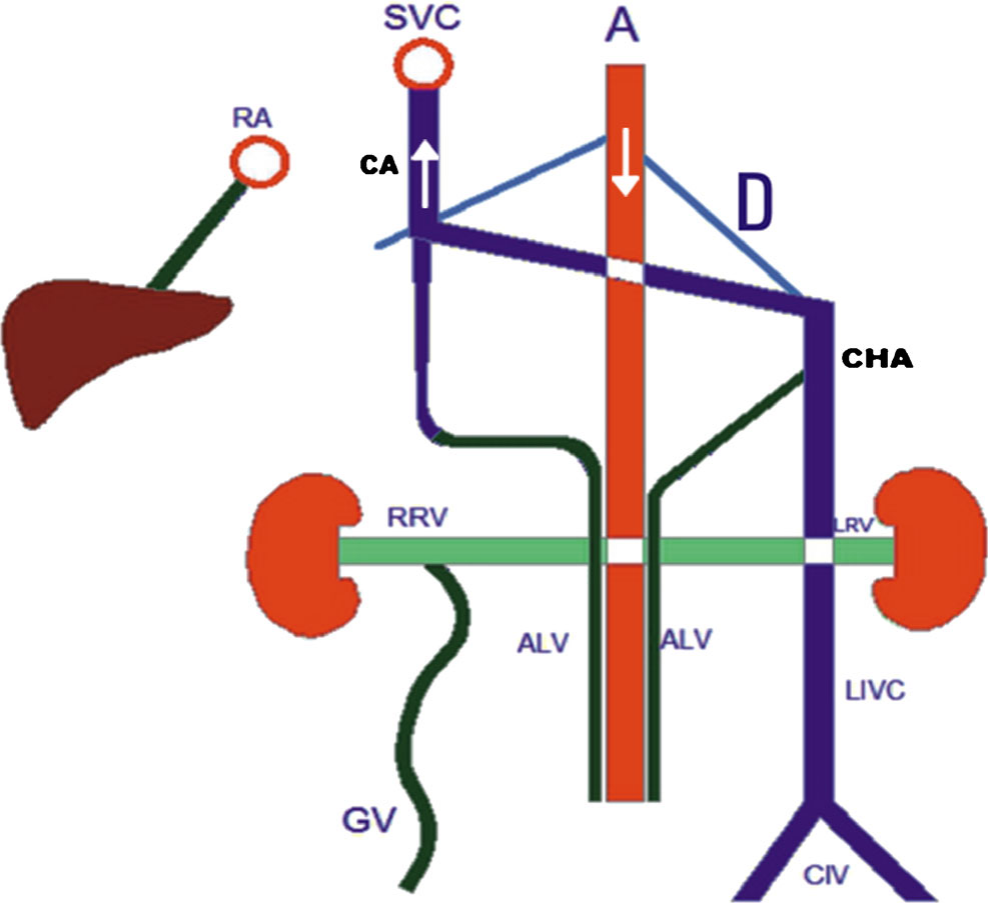
Illustration of findings of left IVC (LIVC) with continuous azygos (CA) and hemiazygos (CHA) veins. Note drainage of a dilated gonadal vein (GV) to the right renal vein (*median collateral pathway*). Ascending lumbar veins (*deep pathway*) are draining into CA and CHA. (RA: right atrium; A: aorta; SVC: superior vena cava; RRV and LRV: right and left renal veins, respectively; CIV: common iliac veins; D: diaphragm)

**Fig. 8:**
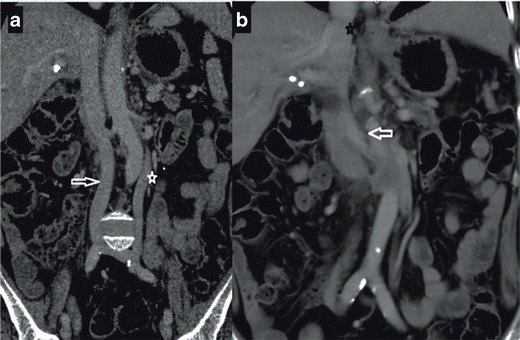
**a** Coronal MIP MDCT image showing parallel elongated segments of CHA(*white arrow*) and CA (*black arrow*) with concurrent right (*asterix*) and left IVC (*arrow head*) in the infrarenal segment **b** Axial MIP MDCT image showing left IVC (*arrow head*) draining into the LRV (*white arrow*)and the right IVC (*black arrow*) into the RRV (*black asterix*) at the renal level **c** Coronal MIP MDCT image showing hemiazygos vein (*white arrow*) joining azygos (*White asterix*) at the level of T6 and its progress

In the second of our cases with a left IVC, RRV was not retroaortic but passes anterior to the aorta and draining into the left IVC (Fig. 6b). Finally, in our third patient, in addition to a left IVC that was continuous with CHA and CA (Fig. 4), a retroaortic RRV was draining into the left IVC. The deep pathways we mentioned previously were open in all these three patients (Figs. 7 and 8).

The deep pathways we mentioned previously were open in all these three patients with left IVC.

### Double IVC

Typical double IVC has a prevalence of 0.2 %-3 % [2, 3]. Double IVC is caused by persistence of both the left and right supracardinal veins. The left IVC may cross over and join the right IVC. In some patients, remarkable differences may be present regarding the size of the left and right IVC. This anomaly can also be associated with other variations including right double IVC, double IVC with the retroaortic RRV, and double IVC with CHA of the left IVC [2, 3, 5, 7, 9].

Double IVC with retroaortic RRV and CHA of the IVC can occur in three patterns: (1) hemiazygos vein joins the rudimentary azygos, (2) hemiazygos vein joins the coronary vein of the heart through a persistent left SVC, and (3) accessory hemiazygos vein progresses to the left brachiocephalic vein [2, 3].

**Fig. 9:**
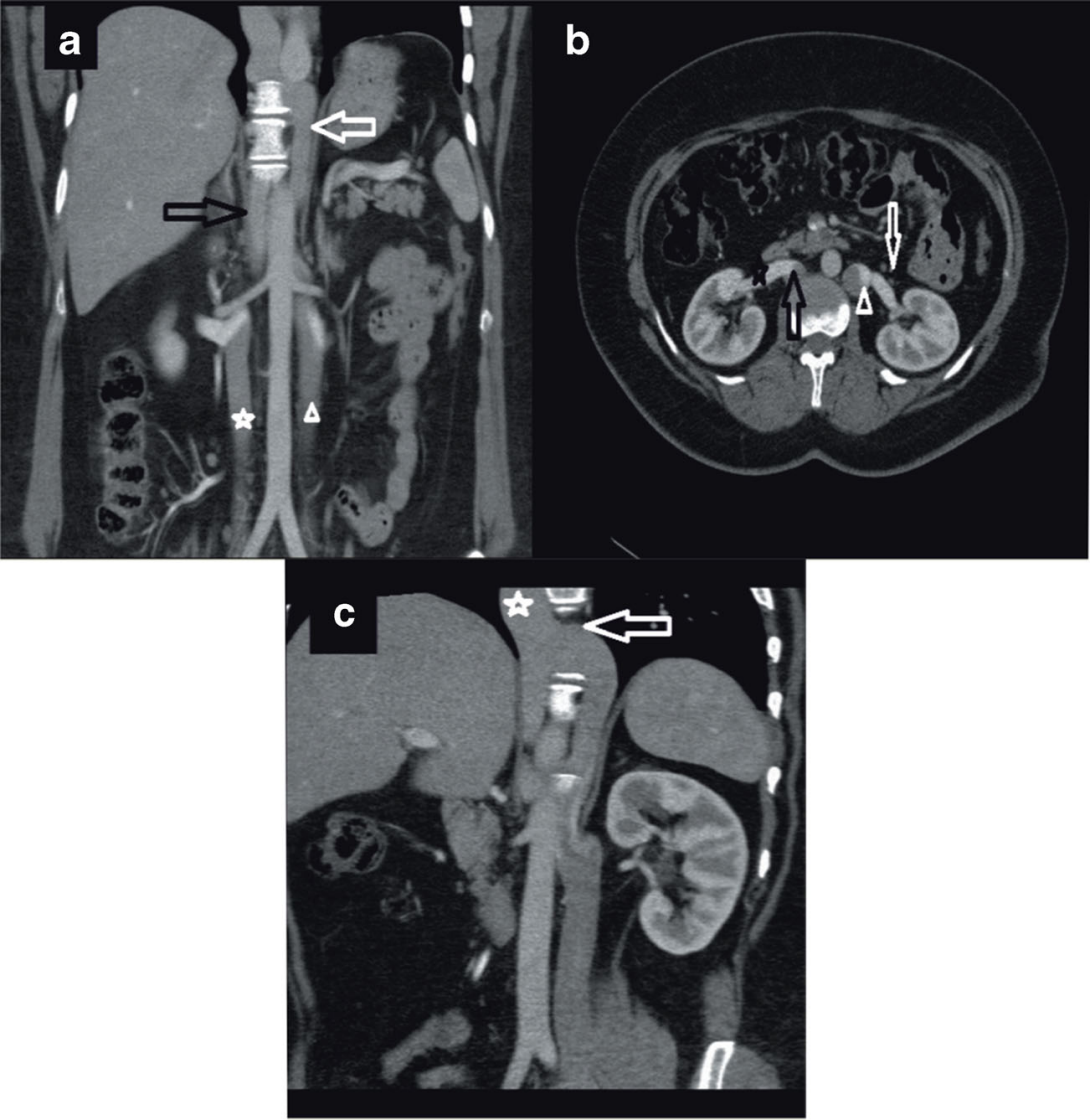
**a** Coronal MIP MDCT image showing concurrent appearance of right (*arrow*) and left IVC in the infrarenal segment (*asterix*) **b** Coronal MIP MDCT image showing left IVC joining the right IVC (*arrow*) at the renal level and right IVC draining into the right atrium following the intrahepatic segment (*asterix*)

**Fig. 10:**
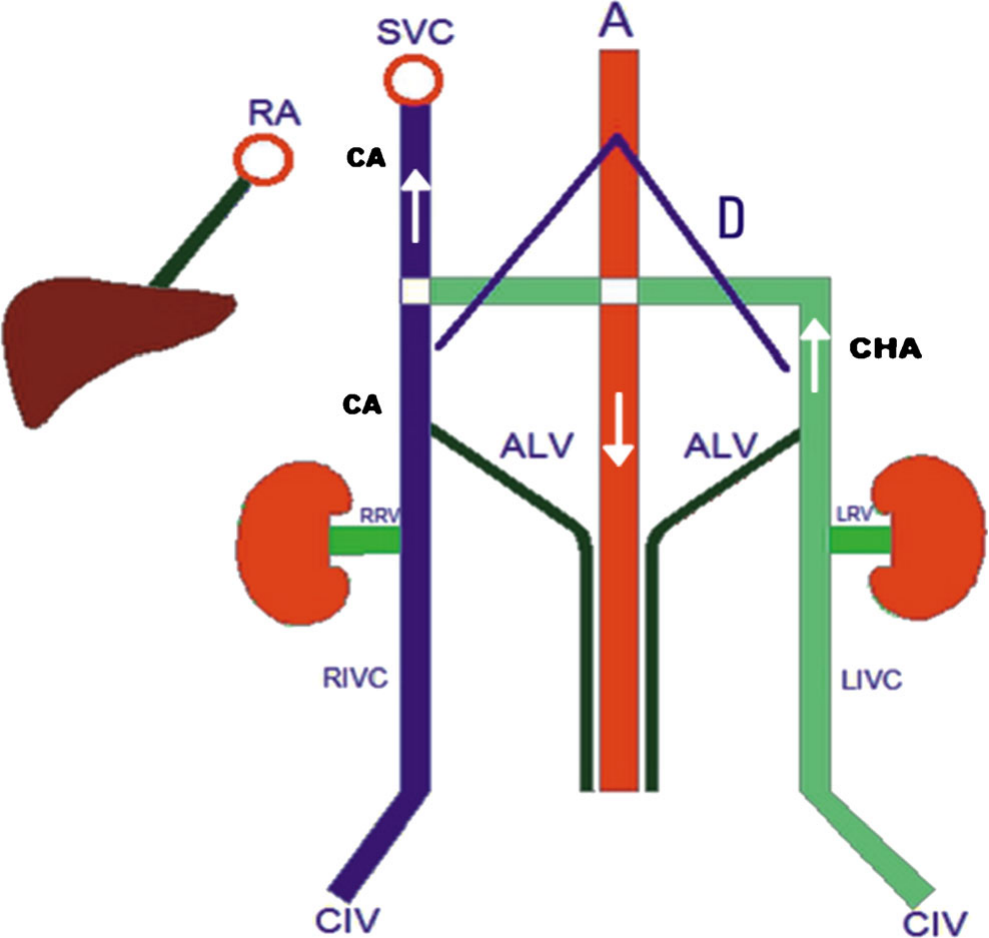
Illustration of findings of double IVC. Note that the right renal vein (RRV) and left renal vein (LRV) drain to the right inferior vena cava (RIVC) and the left inferior vena cava (LIVC), respectively. The continuous hemiazygos vein (CHA) joins the continuous azygos vein (CA) suprediaphragmatically. Ascending lumbar veins drain to the CA and the CHA (deep collateral pathway). (RA: right atrium; A: aorta; SVC: superior vena cava; CIV: common iliac vein; D: diaphragm)

We encountered two cases with double IVC. In the first case, typical double IVC was present (Fig. 9). In the second case, the right IVC was continuous with the azygos vein, and the left IVC was continuous with the hemiazygos vein; continuous azygos and hemiazygos veins joined together supradiaphragmatically and drained into the SVC (Fig. 9). The portal system was normal and the deep pathways were being used. To the best of our knowledge, no other case with such an elongated hemiazygos vein and a supradiaphragmatic combination of azygos and hemiazygos veins were reported previously. Figure 10 illustrates the imaging findings of double IVC.

**Fig. 11:**
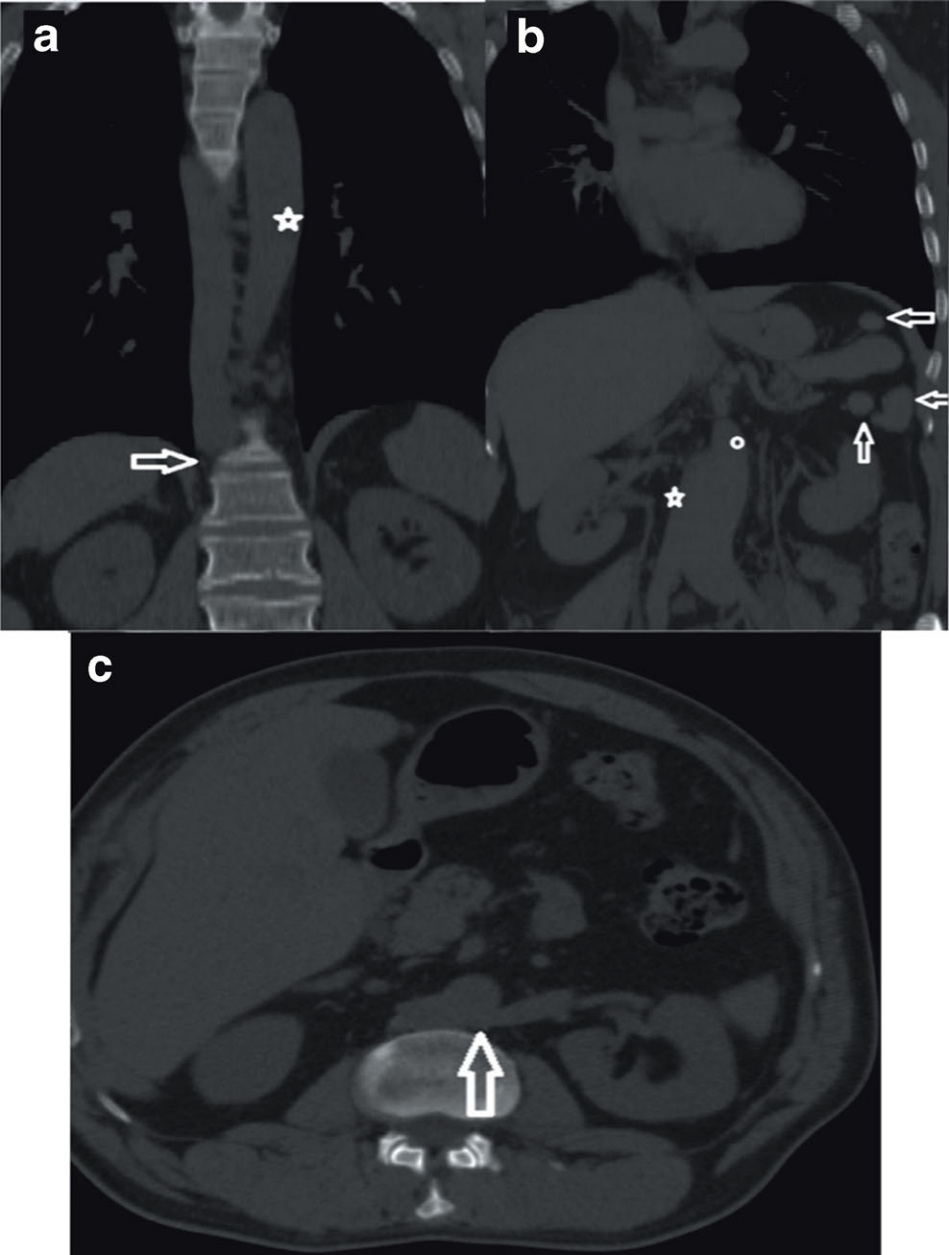
**a** Coronal MDCT image showing interruption of the IVC and its continuation with azygos vein (*arrow*); aorta is marked with an asterix **b** Coronal MPR MDCT image showing normal IVC (on the *right*) (*asterix*) with associated splenosis (*arrows*); aorta is marked with a small white circle **c** Axial MDCT image showing retroaortic LRV joining the right IVC (*arrow*)

**Fig. 12:**
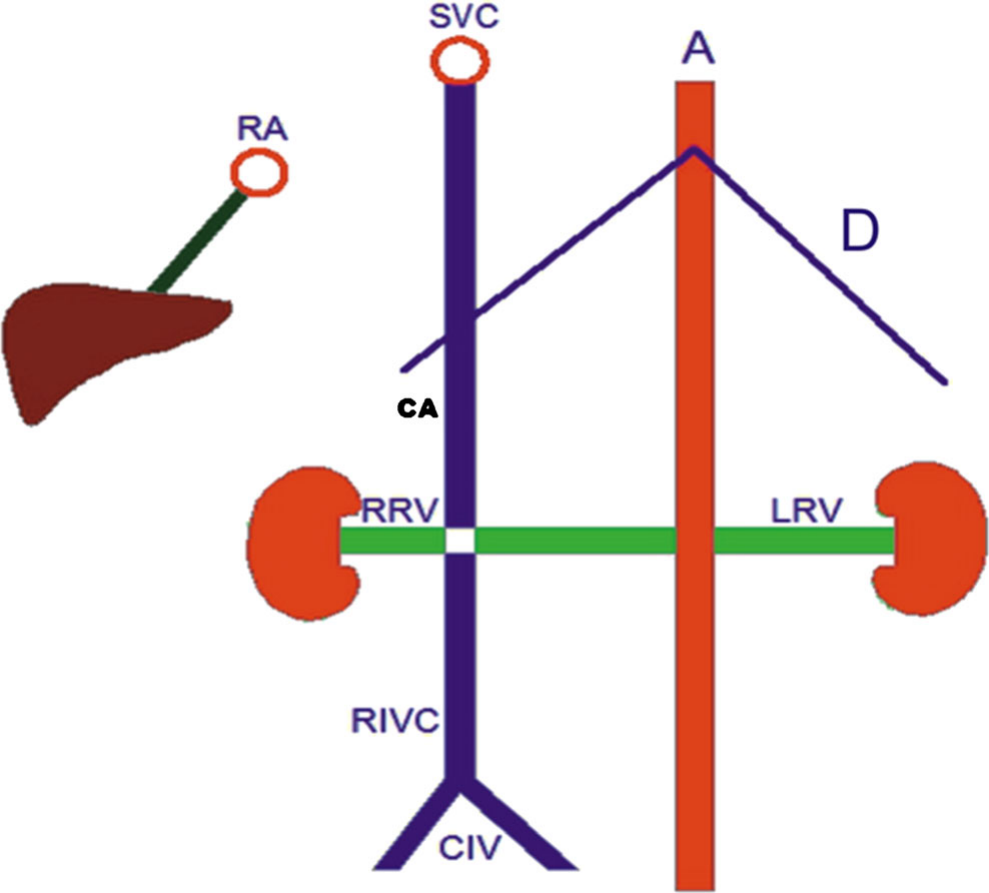
Illustration of findings of interrupted IVC (RIVC) and its continuation with azygos vein (CA). Right renal vein (RRV) and retroaortic left renal vein (LRV) drain to CA (RA: right atrium; A: aorta; SVC: superior vena cava; CIV: common iliac vein; D: diaphragm)

Infrahepatic interruption of the IVC is a relatively rare anomaly with CA, having a prevalence of 0.6 % [2, 3, 7]. Although it sometimes occurs as an isolated entity, it is more frequently associated with other cardiovascular malformations and situs anomalies, such as the polysplenia syndrome [10]. This anomaly is caused by a failure in the development of the right subcardinal-hepatic anastomosis with subsequent atrophy of the suprarenal IVC [3]. In patients with this anomaly, the infrarenal IVC continues as the azygos vein and, if there is a left sided IVC, it continues as the hemiazygos vein. The case we present in this paper shows typical findings of this entity (Fig. 11): an interruption of the infrarenal inferior vena cava and its continuation as the azygos vein. The patient showed signs of polysplenism, as well (Fig. 11b, arrows). Figure 12 illustrates vascular imaging findings of interrupted IVC with azygos continuation.

**Fig. 13:**
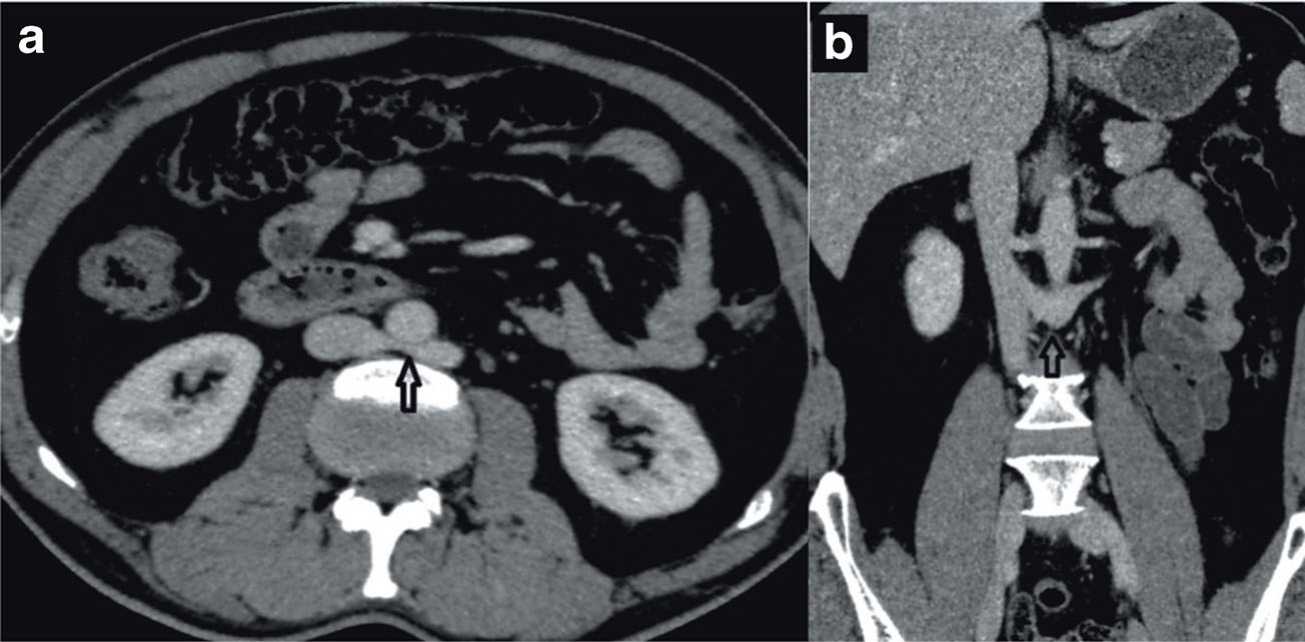
**a** Axial MDCT image showing retroaortic LRV Type I joining the IVC **b** Coronal MDCT image showing left retroaortic vein joining the IVC (*black arrow*)

In cases with a retroaortic left renal vein, the anterior subcardinal veins regress completely, and only the retroaortic supracardinal veins remain to maintain the connection of the left kidney to the inferior vena cava [21]. A retroaortic left renal vein is the most common form of IVC anomalies with a prevalence of 2.1-3.4 % [2, 3]. In this paper we present a typical case with a left retroaortic renal vein. In our case LRV was crossing the retroaortic area to join the right IVC, and the IVC was normal (Fig. 11). Interestingly, our patient was presented with chronic epigastric and abdominal pain with no positive imaging or clinical findings other than LRAV (Fig. 13).

### Isolated accessory hemiazygos vein

**Fig. 14:**
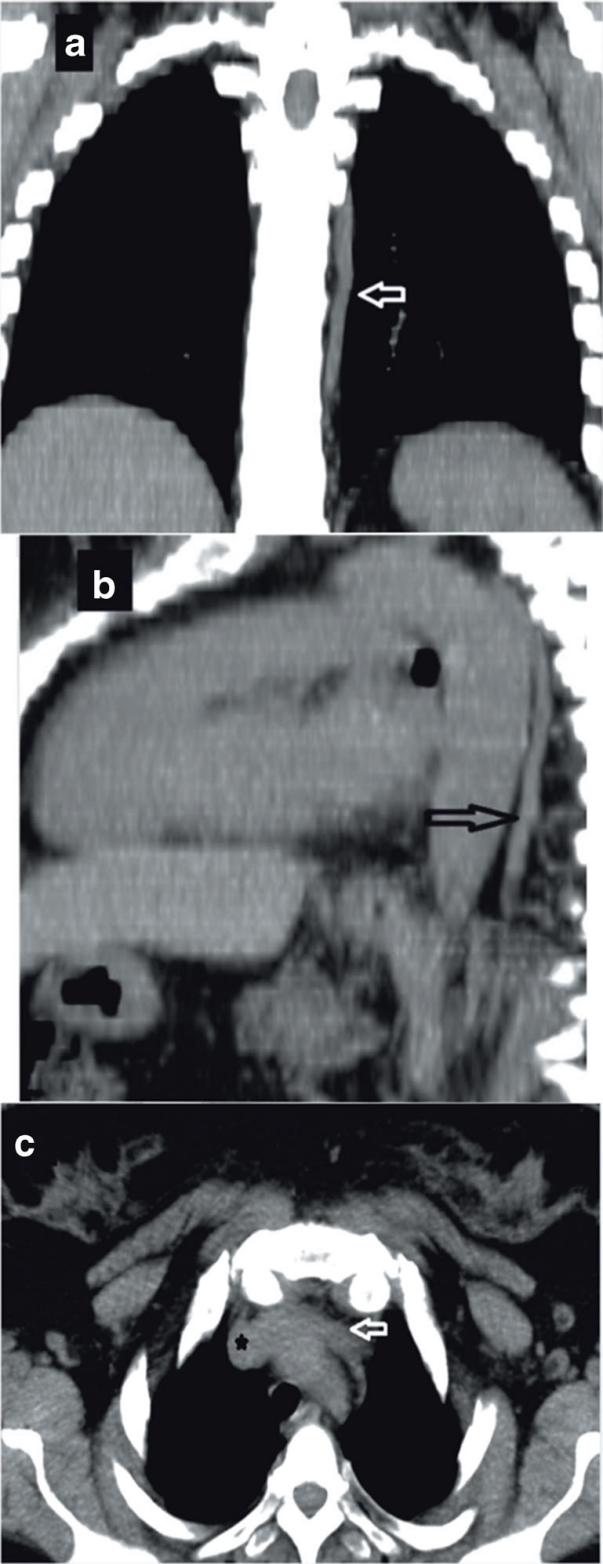
**a** Coronal MDCT MPR image showing accessory CHA progressing in the left paramedian area (*arrow*) **b** Sagittal MDCT MPR image showing accessory CHA (*arrow*) **c** Axial MDCT MPR image showing the segment of accessory CHA (*arrow*) joining the brachiocephalic trunk (*asterix*)

Our last patient had a unique anomaly of IVC, which was an isolated accessory continuous hemiazygos vein. To the best of our knowledge, no other case with the same isolated anomaly was published previously. The patient we present in this paper was admitted to the hospital because of vague chest pain. His x-ray revealed sequelae of pulmonary tuberculosis in the upper lobe of the right lung. A CT scan demonstrated an accessory CHA, which was draining into the brachiocephalic vein on the left side. On the right side, a normal IVC and normal right azygos vein were draining into the SVC, as usual (Fig. 14). Improvements in MDCT and MRI technologies have enabled prompt and correct diagnosis of vascular anomalies associated with other anomalies and diseases, which also simplified the diagnostic and treatment procedures both for the patients and for the clinicians. IVC anomalies should be suspected in the patients presenting with pulmonary emboli, chronic pain, and DVT. Awareness of IVC anomalies and variations is crucial both for clinical and surgical procedures, and unawareness of these anomalies may lead to severe and deadly complications.
